# Dihydroquercetin ameliorates LPS-induced neuroinflammation and memory deficit

**DOI:** 10.1016/j.crphar.2022.100091

**Published:** 2022-02-10

**Authors:** Qadir Alam, Sairam Krishnamurthy

**Affiliations:** Neurotherapeutics Laboratory, Department of Pharmaceutical Engineering & Technology, Indian Institute of Technology (Banaras Hindu University), Varanasi, 221005, U.P, India

**Keywords:** Dihydroquercetin, Neurodegenerative disease, Learning and memory, Neuroinflammation, Intracerebroventricular injection, LPS, Lipopolysaccharide, ROS, Reactive oxygen species, DHQ, Dihydroquercetin, Ach, Acetylcholine, AchE, Acetylcholine Estrase

## Abstract

Dihydroquercetin (DHQ) is a pentahydroxyflavanone that has been used as an important suppliment against oxidative stress related inflammation and neuroinflammation. Neuroinflammation, which is the activation of the defense mechanism of the central nervous system, upon exposure to stimuli like amyloid β, Lewy bodies, lipopolysaccharide (LPS) and reactive oxygen species. It is an important pathophysiological mediator of a number of neurodegenerative disorders, including Alzheimer's disease, Parkinson's disease, multiple sclerosis and others. The objective of the present study is to evaluate the neuroprotective effect of DHQ, a potent antioxidant molecule, against LPS induced neuroinflammation. On the first day of the experiment (day-1), neuroinflammation was induced through intracerebroventricular injection of LPS (5 ​μg/5 μl) into each lateral ventricle in the rats. DHQ-0.5, 1 and 2 ​μg/kg was injected into the tail vein in respective groups from day-2 to day-10. Behavioral studies showed that DHQ attenuated the LPS-induced loss in long-term memory and working memory as evaluated by elevated plus maze and Y-maze test, respectively.

Further, the biochemical estimations revealed that DHQ dose-dependently attenuated the LPS-induced decrease in acetylcholine level and increased in the acetylcholine-esterase activity in the hippocampal region. DHQ also increased the catalase activity and decreased nitric oxide and lipid peroxidation altered by LPS injection. DHQ also attenuated interleukin-6 in the brain, which has elevated upon LPS induction. The decrease in IL-6 is attributed to its antioxidant activity. Hence, DHQ could be a potential therapeutic candidate in the management of neuroinflammation and related neurodegenerative disorders.

## Highlights

• Dihydroquercetin (DHQ) improves LPS induced loss in working memory and long-term memory.
• DHQ attenuates LPS –induced decrease in cholinergic activity.
• DHQ shows anti-oxidant properties.
• DHQ shows anti-neuroinflammatory effect.

## Graphical abstract

**Figure:**
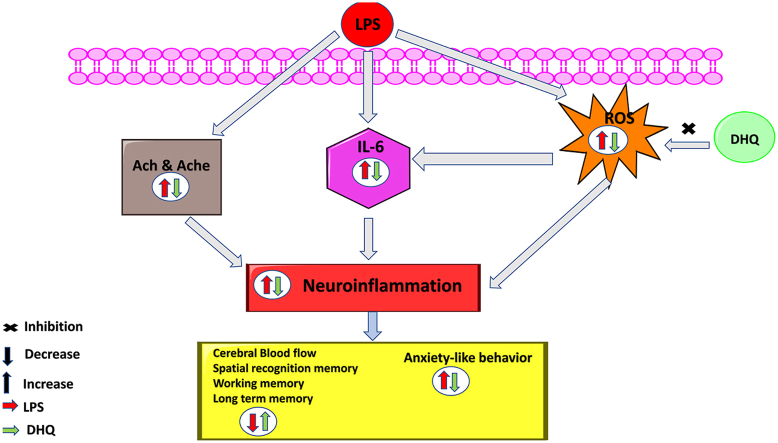

## 1 Introduction

Neuroinflammation is the phenomenon of activation of a defense mechanism in the central nervous system against a variety of stimuli (Morales et al., 2015). It is an important pathophysiologic mediator of most of neurodegenerative disorders including Alzheimer's disease, Parkinson's disease, multiple sclerosis, and others (Lyman et al., 2014; Morales et al., 2015). Various stimuli include amyloid β, Lewy bodies, lipopolysaccharide (LPS), reactive oxygen species (ROS), are crucial mediators of neuroinflammation (Lee et al., 2008; Streit et al., 2004). LPS is an established model for studying the relation between neuroinflammation and cognitive behavior (Tripathi et al., 2017). LPS is also reported to induce anxiety-like behavior in mice and rat models (Bassi et al., 2012; Savignac et al., 2016).

Although the exact pathophysiology of neuroinflammation by LPS is not completely known, one of the mechanisms is toll like receptor (TLR)-4 mediated activation of NFκB, which is a transcription factor for many pro-inflammatory cytokines (Shih et al., 2015). Important mechanism for neuroinflammation by LPS is cellular damage due to reactive oxygen species (ROS) generation and cytokines activation (Hsu and Wen, 2002). LPS induces activation of astrocytes and microglia in the CNS and a further increase in the expression of IL-6 (Wang et al., 2015). IL-6 is a soluble mediator with a pleiotropic effect on inflammation, immune response, and hematopoiesis and is considered as a marker of neuroinflammation when present in CNS (Beurel and Jope, 2009). LPS causes neurodegeneration and defects in learning and memory (Shaw et al., 2001). It is proven that both muscarinic and nicotinic acetylcholine receptors play a role in learning behavior and memory consolidation via long term potentiation (LTP) (Hasselmo, 2006). As per the cholinergic hypothesis, loss of cholinergic function in the CNS is significantly related with the memory decline and acetylcholine esterase inhibitors are pivotal drugs for the management of dementia related symptoms in Alzheimer's disease (Davies, 1985).

Neuroinflammation is critically involved in neurodegenerative disorders; however, the conventional anti-inflammatory drugs, such as non-steroidal anti-inflammatory drugs, have produced mixed results, and their toxic effects have not been resolved (Woodling and Andreasson, 2016). Therefore, there is a need to explore newer drugs for the potential treatment of neuroinflammatory disorders. There are various natural products showing promising results against oxidative stress and neuroinflammation, including carnosine (Caruso et al., 2019), hederagenin (Wang et al., 2020), resveratrol (Zhang et al., 2013).

Dihydroquercetin (DHQ) is a potent antioxidant flavonoid found in onions, French maritime bark, milk thistle, Douglas fir bark (Weidmann, 2012). It has been found to possess a neuroprotective effect on rat neuronal culture cells which were damaged by oxidative stress (Dok-Go et al., 2003). It has been reported to ameliorate concanavalin-A-induced mouse experimental fulminant hepatitis and increased heme oxygenase-1 (HO-1) expression through mitogen-activated protein kinase/nuclear factor erythroid 2-related 2 (MAPK/Nrf2) antioxidant pathway in RAW264 macrophage cell lines (Zhao et al., 2015). DHQ has also shown to inhibit cerebral ischemia-reperfusion injury in rats through suppression of leukocyte infiltration and by inhibiting cyclooxygenase-2 and the expression of inducible nitric oxide synthase (Wang et al., 2006). DHQ has also attenuated the proteasome inhibition-induced apoptosis in PC12 ​cells by suppressing the activation of the mitochondrial pathway and caspase-8 and Bid-dependent pathways through antioxidant action (Nam et al., 2015). Therefore, we thought it would be prudent to evaluate the anti-neuroinflammatory activity of DHQ as it possesses potent antioxidant activity.

Therefore, the objective of the present study is to evaluate the potential anti-neuroinflammatory effect of DHQ (0.5, 1 and 2 ​μg/kg) in the LPS model in rats. Functional analysis of DHQ treatment was done by estimating cerebral blood flow and behavioral tests for working and long-term memory apart from anxiety-like effects using Y-maze and elevated plus maze test, respectively. The level of acetylcholine (ACh) and acetylcholine esterase (AChE) activity were investigated in the hippocampus region to further evaluate learning and memory. Anti-neuroinflammatory activity of DHQ was evaluated by estimating expression of IL-6.

## 2 Materials and methods

### 2.1 Experimental animals

Inbred albino Wistar male rats of weight (260 ​± ​20 ​g) were procured from Central Animal House; Institute of Medical Science (IMS-BHU). The experiments were performed by adopting guidelines (NIH publication number 85–23, revised 2011) and approved by the Institutional Animal Ethical Committee, Banaras Hindu University (BHU; Dean/2015/CAEC/1420). Animals were acclimatized for one week in the experimental lab before initiating the experiments.

### 2.2 Chemicals

Dihydroquercetin (Disto-Pharmaceuticals, India), LPS (*E. coli*, L3129), bovine serum albumin, and griess reagent were procured from Sigma-Aldrich (St. Louis, MO, USA). thiobarbituric acid (TBA), NADH, sodium succinate, sodium azide, phenazinemethanesulphonate (PMS) and nitro blue tetrazolium (NBT) were purchased from Merck (Darmstadt, Germany). All other chemicals and reagents of high-performance liquid chromatography (HPLC) and analytical grade were procured from local suppliers.

### 2.3 Experimental procedure

**Fig. 1:**
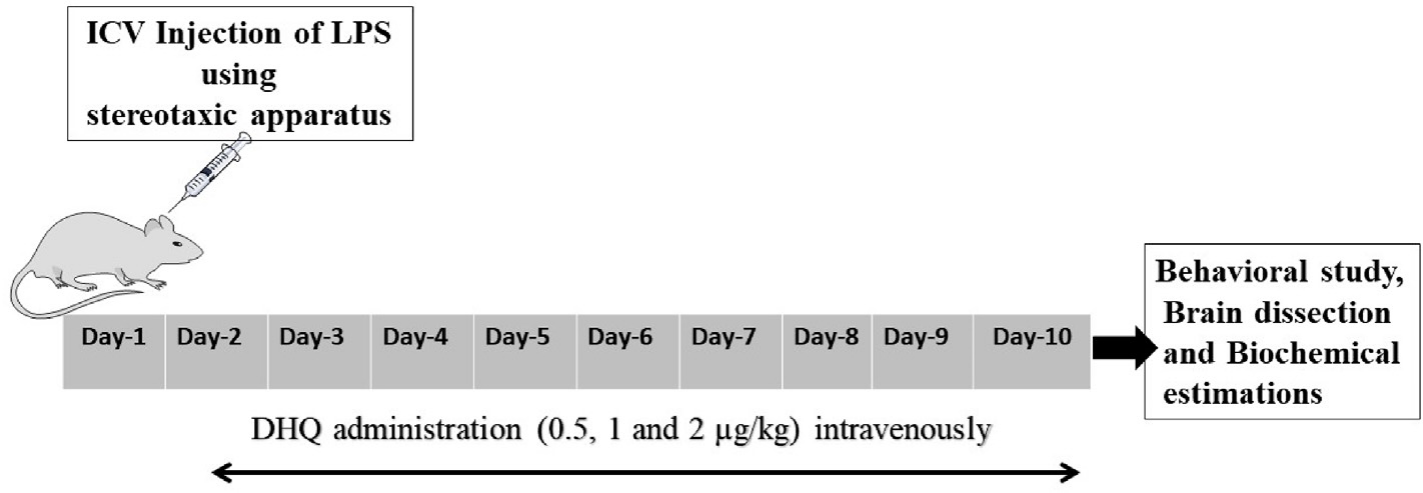
The schematic representation of the experimental schedule. Briefly, in the experiment, lipopolysaccharide (LPS) was administered to rats on day-1 of the design. From day-2 onwards, Dihydroquercetin (DHQ) was administered for nine days.

The schematic representation of the experimental schedule has been depicted in Fig. 1. The LPS was injected intracerebroventricular (ICV) using stereotaxic apparatus (Stoelting, USA). Rats were anaesthetized with sodium pentobarbitone; i. p. 45 ​mg/kg (Tripathi et al., 2017). The animal was fixed on the stereotaxic frame, and the scalp of the rat was incised, and bregma was positioned on the scalp of the rat. All coordinates were set from the bregma (0, 0) and drilled 0.8 ​mm posterior to bregma, 1.5 ​mm lateral to sagittal suture and 3.8 ​mm beneath the surface of the brain (Hauss-Wegrzyniak et al., 1998). LPS was administered to all the groups except sham by using Quintessential Stereotaxic Injector. LPS solution was injected into each of the lateral cerebral ventricles at concentrations of 1 ​μg/μl, at a rate of 1 ​μl/min over a period of 5 ​min with a waiting period of 5 ​min between the two injections. Sham group was also exposed to surgery, and only saline (vehicle) was injected. All the sham group results were not significantly different from the control group, so the group was not included further. To limit local infection, after suturing up the incision, we have applied betadine (iodine solution) up to day-5, 1 ​ml of normal saline was injected intraperitoneally to prevent dehydration in the animals (Jangra et al., 2021). Rats were closely monitored during the recovery period and kept in a room at 22–26 ​°C. This was considered as day-1, and after 24 ​h, upon the appearance of neuromotor dysfunction related symptoms, the drugs were given daily for nine days. All the behavioral tests were performed on day-10 and recorded using ANY-MAZE behavioral tracker version 4.72 (USA). The animals were then immediately killed by decapitation and, hippocampus tissues were microdissected (Paxinos and Charles, 2006). The brain tissues were stored at −80 ​°C until further mechanistic studies.

### 2.4 Drug administration

DHQ was first dissolved in 10 ​μl of ethanol, and then the volume was made up using normal saline to obtain a concentration of 1 ​mg/ml. The concentration of ethanol in the solution injected was less than 0.001% (v/v) and the same concentration excluding DHQ was administered to vehicle control group. Previously, it has been reported that DHQ ameliorates cerebral ischemia-reperfusion injury in rats through its anti-oxidative effect and modulation of NFκB activation at the dose of 0.1 and 1.0 ​μg/kg, intravenous (IV) daily (Wang et al., 2006). Therefore, we conducted a pilot study to evaluate the DHQ IV dose and finalized three doses of DHQ 0.5 ​μg/kg, 1 ​μg/kg, and 2 ​μg/kg.

By following the principle of randomization, grouping was done taking nine animals in each group as follows: (a) Control, (b) LPS (c) LPS ​+ ​DHQ 0.5 ​μg/kg, (d) LPS ​+ ​DHQ 1 ​μg/kg, (e) LPS ​+ ​DHQ 2 ​μg/kg. There were no *par se* group in the experiment to check the effect of DHQ alone on the rats as the toxic and therapeutic effects of DHQ are well known (Booth and DeEds, 1958; Sunil and Xu, 2019).

### 2.5 Measurement of cortical blood flow

CBF were recorded using laser speckle blood flow imaging system (omegazone OZ-2 STD, Tokyo, Japan). Rats were anaesthetized, and their skull bones were exposed by a midline scalp incision and placed on the black sponge sheet under the arm stand. The arm stand holds the CCD camera, the lens (ZM10–18, MF12), and the laser unit (780 ​nm for measurement and 650 ​nm for positioning). Raw speckle images were recorded from the skull surface using LSI Software (LSI ver.3.3, Omegawave, Inc., Tokyo, Japan), and average cerebral blood flow was determined by further analysis of images by using LIA Software (LIA ver.3.3, Omegawave, Inc., Tokyo, Japan). The black sheet does not reflect the laser light, and the effect makes the blood flow image clear (Paliwal et al., 2018).

### 2.6 Behavioral tests

#### 2.6.1 a Y-maze test

Y-maze apparatus helps to assess the working memory, general exploratory behavior and spatial memory. The Y-maze apparatus is made up of three identical arms having dimensions of 32 ​cm height, 50 ​cm length, and 16 ​cm width, which are angled at 120⁰ to each other. Plastic balls of different colors were placed around the arms, and these were not changed every time before the test to maintain novelty for the animals. Soiled animal bedding was spread over the floor of the maze to give a homely atmosphere. In trial 1, the novel arm of the Y-maze was kept closed, and the animals were free to move in the two arms for 15 ​min. Trial 2 was performed exactly after 4 ​h of trial 1, in which animals had free access for 5 ​min to all three arms. All the entries were recorded using ANY MAZE software. Curiosity behavior was calculated from a total number of entries in all three arms. The percentage of arm entries in novel and known arms is considered as indicative of spatial recognition memory. Spontaneous alternation behavior signifies the working memory in which we observe how often an animal repeats its initial choices of arm entries. An arm entry is counted when the animal has his head and front paws inside an arm (Garabadu et al., 2015; Joshi et al., 2014).

#### 2.6.2 b elevated plus maze test

The EPM (Elevated Plus Maze) test was performed to assess anxiety and long-term spatial memory behavior in a fabricated apparatus. The fabricated EPM consisted of two open arms (50 ​× ​10 ​cm) and two closed arms (50 ​× ​10 ​× ​40 ​cm) the height of the open arm roof was 50 ​cm from the floor. Transfer latency (TL) was performed for evaluating long-term spatial memory. TL was measured as the time taken by the animal to move into one of the enclosed arms with all its four legs. The animal was gently pushed into one enclosed arm if the animal is not entering into the enclosed arm within 90 ​s. In such a case, TL was counted as 90 ​seconds. On day-10, the trial was performed in which rats were allowed to explore the maze for 2 ​min. This task was repeated after 24 ​h to evaluate the retention of memory. Percentage entries and time spent in the open arm was considered as a measure of anxiety (Krishnamurthy et al., 2013).

### 2.7 Biochemical estimations

#### 2.7.1 a preparation of the samples

The hippocampus tissue homogenate was made, taking hippocampus tissue in 1 ​ml of 0.1 ​M perchloric acid in a Potter–Elvehjem homogenizer with fine circular unidirectional trituration. The homogenate so obtained was taken in a polypropylene tube in which 50 ​μl of 4 ​M potassium acetate was added to make its pH 4.0, and later, it was centrifuged for 15 ​min at 4000 ​g. The supernatant so obtained was used for the estimation of both Acetylcholine (Ach) and Acetylcholine Esterase (AchE) activity (Muthuraju et al., 2009).

#### 2.7.2 Spectrofluorometric assay of acetylcholine

Amplex red assay kit was used to find the amount of acetylcholine in the homogenate. The reaction was started by adding Amplex Red reagent/HRP/choline oxidase/AChE working reagent in the homogenate tube. It was incubated for 30 ​min, and fluorescence was recorded with the help of a microplate reader (BioTek, Synergy H1M, USA) at 530 ​nm excitation and 590 ​nm emission wavelengths (Zoukhri and Kublin, 2001).

#### 2.7.3 b estimation of AChE activity

Amplex Red AChE assay kit (Molecular Probes, Inc., USA) was utilized to measure AChE activity. The estimation was done as per the manufacturer's instructions. The reagents were added to the sample and were kept for incubation. After incubation, the fluorescence was determined with the help of a microplate reader (BioTek, Synergy H1M, USA) at 530 ​nm excitation wavelength and 590 ​nm emission wavelength.

#### 2.7.4 c nitrite level estimation

Nitrite level was estimated as per the method given by Green (Green et al., 1982). 50 ​μl of the supernatant obtained from brain homogenate was mixed with 5 ​μl of nicotinamide adenine dinucleotide phosphate (NADPH), 10 ​μl of flavin adenine dinucleotide (FAD) and 5 ​μl of nitrate reductase. The mixture was incubated for 1 ​h at 37 ​°C in the dark. Zinc sulfate was added to precipitate the proteins. After centrifuging (6000 ​g), equal volumes of supernatant (100 ​μl) and Griess reagent (100 ​μl) (1:1 mixture of 1% sulfanilamide in 3% orthophosphoric acid and 0.1% naphthyl ethylene diamine) were mixed and incubated for 10 ​min at room temperature in the dark. The plates were then read at 540 ​nm by UV spectrophotometer, and NOx was calculated by using a sodium nitrite standard curve.

#### 2.7.5 d mitochondrial LPO or malondialdehyde (MDA) formation estimation

Mitochondrial MDA content was measured by following the standard protocol (Ohkawa et al., 1979). The homogenate was boiled along with 10% SDS, KCl (1.15%), acetic acid (20%) and TBA (0.8%). After cooling in running water, it was extracted with n-butanol. The organic layer so obtained by centrifuging was measured at 532 ​nm. The concentration of MDA was expressed as micromoles of MDA per milligram of protein.

#### 2.7.6 e assessment of catalase activity

Catalase activity was assayed following the method of Luck (1974). Brain homogenates were centrifuged at 10,000 ​rpm for 10 ​min in an Eppendorf microcentrifuge. The supernatant aliquots were mixed with phosphate buffer and hydrogen peroxide The absorbance was measured at 240 ​nm for 3 ​min at a 30-s interval. From the decrease in absorbance, the enzyme activity was calculated (Correa et al., 2000).

#### 2.7.7 f estimation of IL-6 using ELISA kit

Cytokine IL-6 was estimated in brain homogenate using commercially available ELISA kit for rat IL-6 (NOVEX™, Thermo Fischer, USA). The brain samples were suspended in buffer solution (1% Triton X100, aprotinin 200 U/ml, 0.1 ​mM PMSF, 0.1 ​mM benzethonium chloride, 1 ​mM benzamidine and 1 ​mM EDTA) and then centrifuged at 14,000 ​g for 30 ​min at 4 ​°C and the supernatant was separated for cytokines estimation. The pro-inflammatory cytokine interleukin-6 (IL-6) level in whole brain samples were quantified using ELISA kits as per the manufacturer's instruction.

### 2.8 Statistical analysis

All values are expressed as the mean ​± ​standard deviation (SD). For spatial memory in EPM task, repeated measures two-way ANOVA followed by Bonferroni post-hoc test was performed for transfer latency between day-9 and day-10. Similarly, two-way ANOVA followed by Bonferroni post-hoc test was performed for spatial recognition memory and curiosity behavior test of the animals in the Y-maze to assess the locomotor activity in both probe and test sessions. One-way ANOVA followed by post-hoc Student's Newman–Keuls test was performed for the analysis of all other behavioral and biochemical parameters. Graph Pad Prism version 5 (San Diego, CA) has been used for applying the statistics and groups with p ​< ​0.05 were considered as significantly different.

## 3 Results

### 3.1 DHQ ameliorated LPS-induced alterations in spatial recognition memory behavior test in Y-maze test

**Fig. 2:**
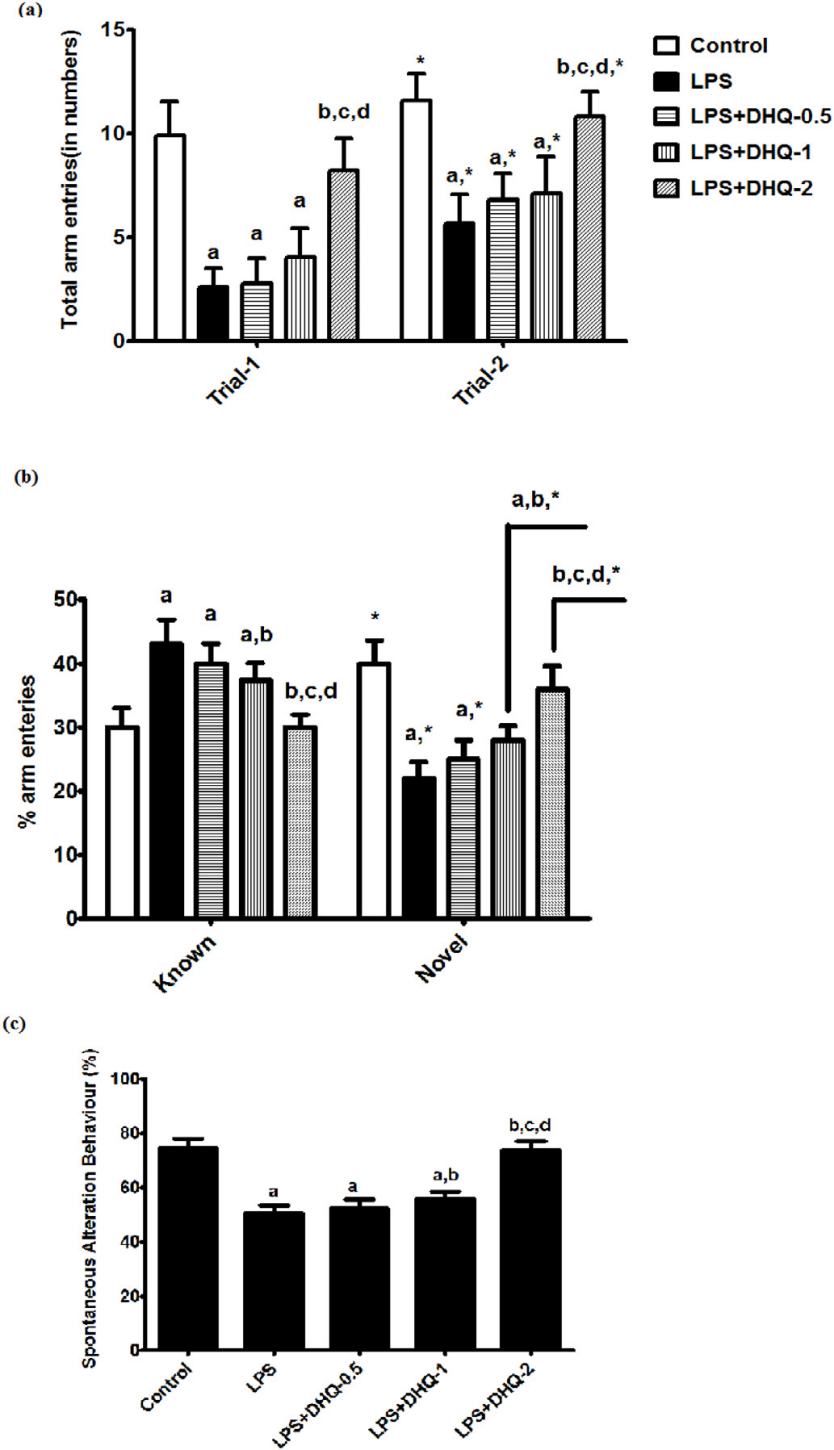
Effect of Dihydroquercetin (DHQ) -(0.5, 1 and 2 ​μg/kg) on LPS-induced alterations in known and novel arm entries (a) and total arm entries in trial-1 and trial-2 (b) and spontaneous alterations (c). in Y-maze test paradigms. All values are Mean ​± ​SD; (n ​= ​9). ^a^p< 0.05 compared to control, ^b^p ​< ​0.05 compared to LPS group and ^c^p ​< ​0.05 compared to LPS ​+ ​DHQ -0.5 group, ^d^p ​< ​0.05 compared to LPS ​+ ​DHQ-1 group, ∗p ​< ​0.05 compared to Known and Trial-1. [one-way ANOVA followed by Student–Newman–Keuls test and two-way ANOVA followed by Bonferroni test].

We performed a spatial recognition memory behavior test in Y-maze to evaluate the effect of DHQ on LPS induced memory deficit behavior. Statistical analysis using two-way ANOVA revealed significant differences in percentage arm entries in trial-1 and trial-2 among groups [F (4, 80) ​= ​2.23, p ​< ​0.05], arm [F (1, 80) ​= ​84.8, p ​< ​0.05] and a significant interaction between groups and arm [F (4, 80) ​= ​88.3, p ​< ​0.05]. Further analysis using post-hoc test showed a significant loss in the spatial recognition memory behavior with LPS administration compared to control rats. There were significant differences among control, LPS, DHQ-0.5 and 1 ​μg/kg groups. Treatment with 2 ​μg/kg attenuated the LPS-induced loss of spatial recognition memory. However, DHQ-0.5 and DHQ-1 μg/kg did not show any change to the LPS-induced loss in the spatial recognition memory. It is shown in Fig. 2(a).

### 3.2 DHQ improved LPS-induced alterations in curiosity behavior test in Y-maze test

We investigated the effect of DHQ on curiosity behavior on day-10 as depicted in Fig. 2(b) below. We found significant differences in trial-1 and trial-2 among groups [F (4, 80) ​= ​1.77, p ​< ​0.05], arm [F (1, 80) ​= ​83.9, p ​< ​0.05] and a significant interaction between groups and arm [F (4, 50) ​= ​99.7, p ​< ​0.05] when analyzed with two-way ANOVA. The post-hoc test revealed that the curiosity behavior was significantly decreased with LPS administration compared to control rats. Treatment of DHQ-2 μg/kg attenuated the LPS-induced decrease in the curiosity behavior. However, DHQ-0.5 ​μg/kg did not cause any change to the LPS-induced decrease in the curiosity behavior. The effect of DHQ-2 μg/kg was found to be more significant than DHQ-1 μg/kg in improving the LPS-induced decrease in the curiosity behavior.

### 3.3 DHQ improved working memory in Y-maze test

The effect of DHQ on spatial memory impairment on day-10 after LPS injection in the Y-maze test is depicted in Fig. 2(c) below. Statistical analysis using one-way ANOVA showed that there were significant differences in spontaneous alteration behavior in Y-maze test paradigm among groups [F (4, 40) ​= ​128, p ​< ​0.05]. The post-hoc test revealed that the spontaneous alteration behavior was significantly decreased with LPS administration compared to control rats. Significant differences were also found between control and DHQ 0.5 and 1 ​μg/kg groups. Treatment of DHQ at 1 ​μg/kg and 2 ​μg/kg attenuated the LPS-induced decrease in the spontaneous alteration behavior, which indicates a gain in working memory. The effect of DHQ-2 μg/kg was found to be more significant than DHQ-1 μg/kg, whereas DHQ-0.5 ​μg/kg did not cause any change to the LPS-induced decrease in the working memory.

### 3.4 DHQ mitigated LPS-induced alterations in transfer latency (TL) in EPM behavior test

**Fig. 3:**
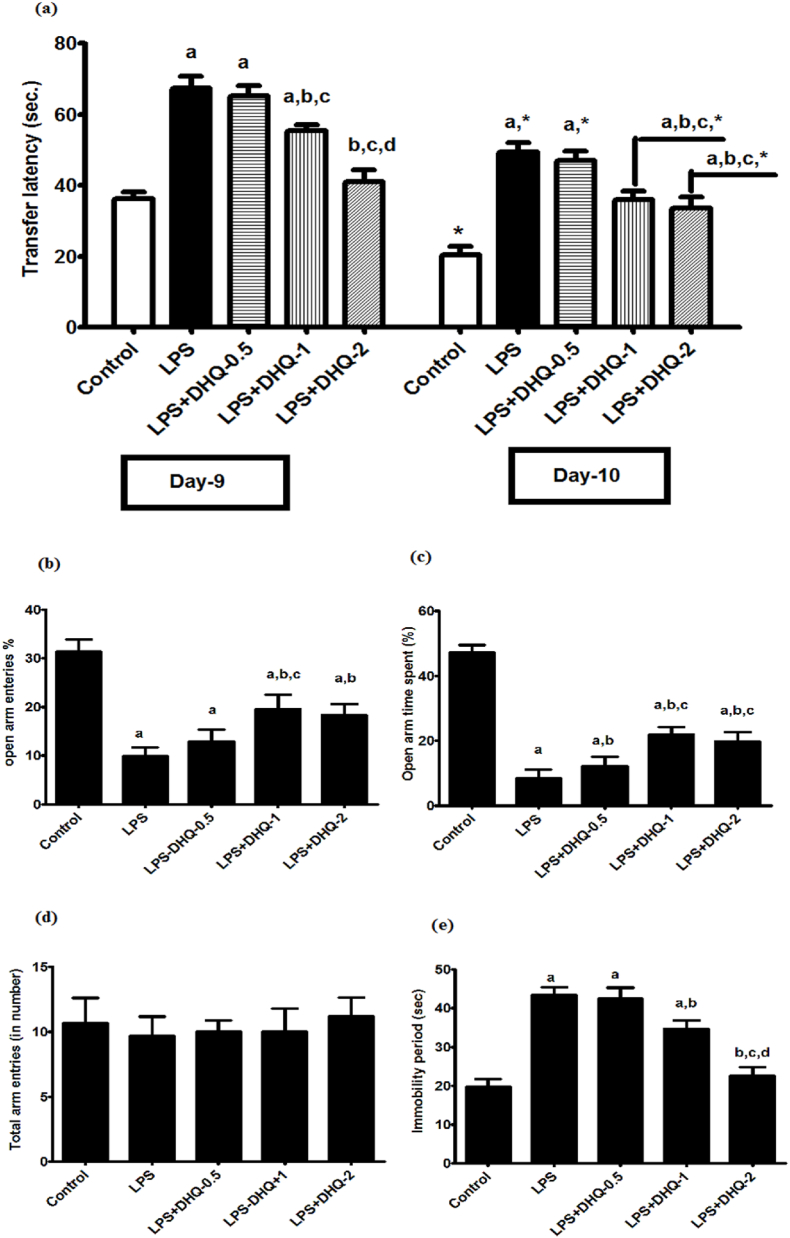
Effect of Dihydroquercetin (DHQ) -(0.5, 1 and 2 ​μg/kg) on LPS-induced alterations in transfer latency (TL) (a) and open arm entries (b) and open arm time spent (c) and total arm entries (d) and immobility period in EPM behavior test. All values are Mean ​± ​SD; (n ​= ​9). ^a^p < 0.05 compared to control, ^b^p ​< ​0.05 compared to LPS group and ^c^p ​< ​0.05 compared to LPS ​+ ​DHQ- 0.5 group, ^d^p ​< ​0.05 compared to LPS ​+ ​DHQ-1 group, ∗p ​< ​0.05 compared to Day-9. [one-way ANOVA followed by Student–Newman–Keuls test and two-way ANOVA followed by Bonferroni test].

In order to evaluate spatial long-term memory, we studied the effect of DHQ on transfer latency on day-9 and day-10 after LPS injection in EPM test as depicted in Fig. 3(a) below. Statistical analysis using two-way ANOVA revealed significant differences in trial-1 and trial-2 among groups [F (4, 80) ​= ​403.1, P ​< ​0.00.05], time [F (1, 80) ​= ​781.4, p ​< ​0.05] and a significant interaction between groups and time [F (4, 80) ​= ​15.11, p ​< ​0.05]. The post-hoc test revealed that LPS injection significantly increased the TL compared to control rats. Data shows that TL of the control group was significantly different from all the other groups on day-10 but on day-9, TL of the control group was significantly different from LPS, DHQ-0.5 and 1 ​μg/kg only. DHQ-0.5 ​μg/kg did not cause any change to the LPS-induced increase in the TL. However, the LPS induced increase in the TL was significantly attenuated with DHQ-1 μg/kg and DHQ-2 μg/kg.

### 3.5 DHQ increased open arm entries in EPM behavior test

Anxiety behavior is caused in rodents by induction of LPS. The effect of DHQ on percentage open arm entries on the day-10 after LPS injection in the EPM test is depicted in Fig. 3(b) below. One-way ANOVA showed significant differences in percentage open arm entries among groups [F (4, 40) ​= ​101, p ​< ​0.05]. The post-hoc test revealed that LPS injection significantly decreased the percentage open arm entries compared to control rats. Data revealed that there were significant differences among control and all other groups. DHQ-0.5 ​μg/kg did not cause any change to the LPS-induced decrease in the percentage open arm entries. However, the LPS induced a decrease in the percentage open arm entries significantly enhanced with DHQ-1 μg/kg and DHQ-2 μg/kg administration.

### 3.6 DHQ alleviated LPS-induced alterations in open arm time spent in EPM behavior test

Behavioral data analysis using one-way ANOVA showed significant differences in open arm time spent among groups [F (4, 40) ​= ​292.2, p ​< ​0.05]. The post-hoc test revealed that LPS injection significantly decreased the percentage open arm time spent compared to control rats. Statistical data also showed that time spent in the open arm by control group was significantly different from all other groups. DHQ-0.5 ​μg/kg caused a significant change to the LPS-induced decrease in the open arm time spent. However, the LPS induced a decrease in the open arm time spent significantly improved with DHQ-1 μg/kg and DHQ-2 μg/kg even more significant than DHQ-0.5 ​μg/kg. It is shown in Fig. 3(c)**.**

### 3.7 DHQ showed no effect in total arm entries in the EPM behavior test

The effect of DHQ on total arm entries on day-10 after LPS injection in the EPM test is depicted in Fig. 3(d) below. Statistical analysis using one-way ANOVA had shown no significant difference in total arm entries among groups [F (4, 40) ​= ​0.894, p ​< ​0.05]. The post-hoc test revealed that LPS injection did not cause any significant changes in total arm entries among the groups. None of the DHQ doses caused any significant changes in the total arm entries.

### 3.8 DHQ dose dependently decreased LPS-induced alterations in immobility period in EPM behavior test

The effect of DHQ on immobility period on day-10 after LPS injection in EPM test is depicted in Fig. 3(e) below. One-way ANOVA revealed significant differences in the immobility period among groups [F (4, 40) ​= ​138.3, p ​< ​0.05]. The post-hoc test showed that LPS injection significantly increased the immobility period compared to control rats. DHQ-0.5 was found to cause no changes in the LPS-induced increase in the immobility period. The immobility period in the control group was significantly lesser than LPS and DHQ-0.5 and 1 ​μg/kg. However, the LPS induced increase in the immobility period was significantly attenuated with DHQ-1 μg/kg and DHQ-2 μg/kg, DHQ-2 μg/kg decreased the immobility period more significantly than DHQ-1 μg/kg.

### 3.9 DHQ improved mean blood flow into the brain altered by LPS induction

**Fig. 4:**
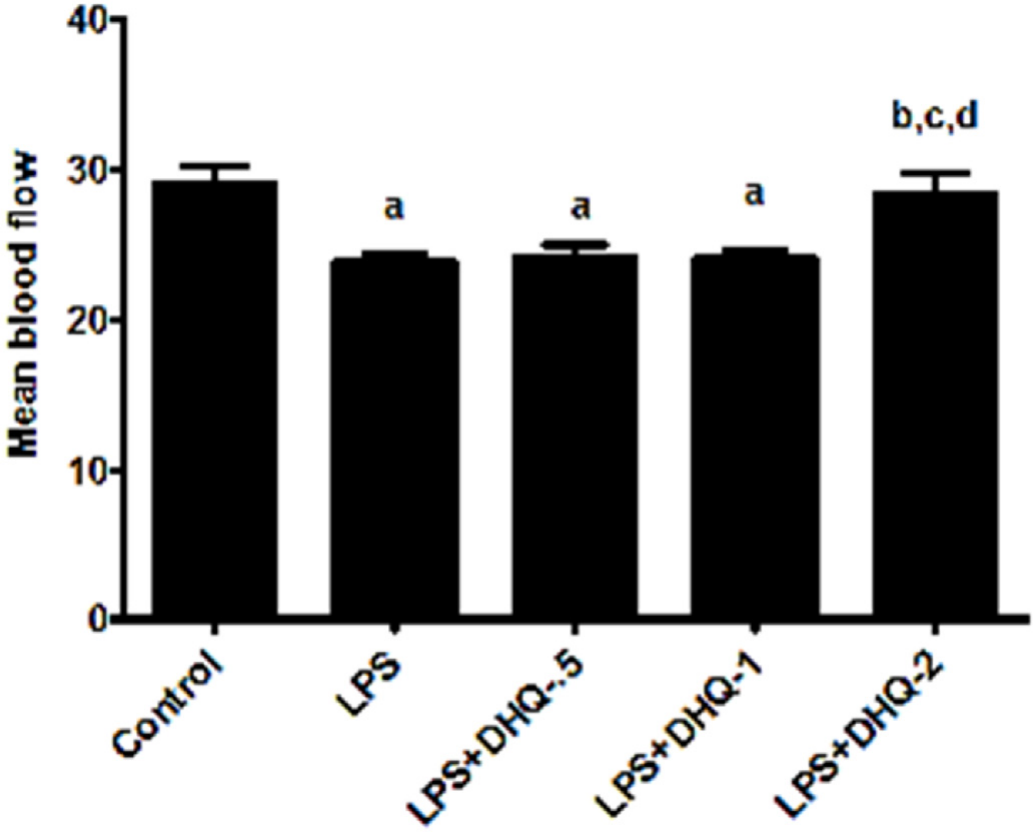
Effect of Dihydroquercetin (DHQ) -(0.5, 1 and 2 ​μg/kg) on LPS-induced alterations on mean cerebral blood flow. All values are Mean ​± ​SD; (n ​= ​6). ^a^p < 0.05 compared to control, ^b^p ​< ​0.05 compared to LPS group and ^c^p ​< ​0.05 compared to LPS ​+ ​DHQ-0.5 group, ^d^p ​< ​0.05 compared to LPS ​+ ​DHQ-1 group [one-way ANOVA followed by Newman-keuls Test].

The effect of DHQ on mean cerebral blood flow was evaluated on day-10 after LPS injection using laser Doppler imager is depicted in Fig. 4 below: Statistical analysis using one-way ANOVA revealed significant differences between the groups [F (4, 25) ​= ​41.9, p ​< ​0.05]. The post-hoc test revealed that LPS injection brought a significant reduction in the mean blood flow into the brain compared to control rats. Blood flow in control rats was also significantly different when compared to DHQ-0.5 and 1 ​μg/kg. However, DHQ-0.5 ​μg/kg and DHQ-1 μg/kg did not cause any change to the LPS-induced decrease in the mean blood flow. However, the LPS induced fall in the mean blood flow was significantly improved with DHQ-2 μg/kg.

### 3.10 DHQ restored the level of acetylcholine altered by LPS induction

**Fig. 5:**
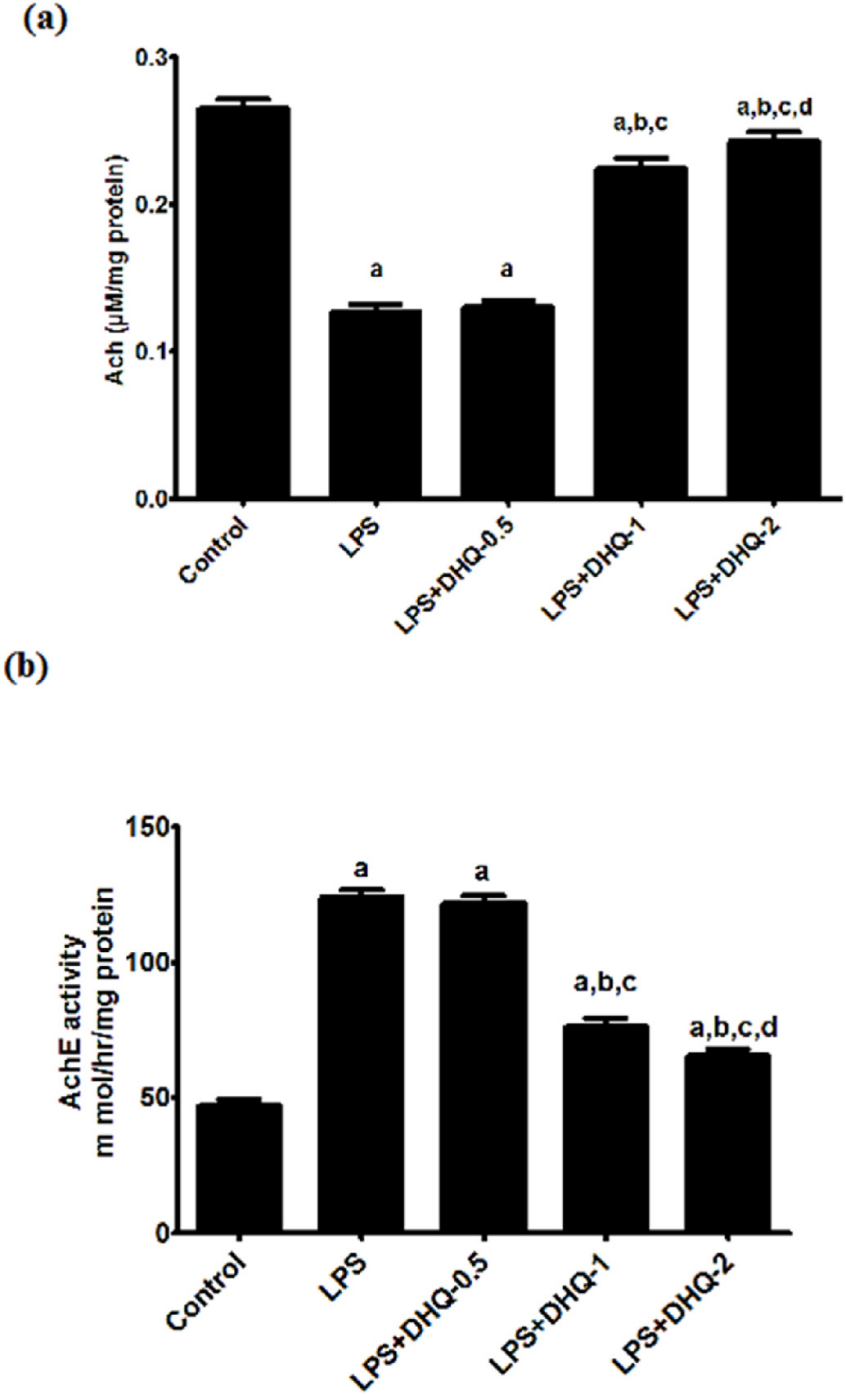
Effect of Dihydroquercetin (DHQ) -(0.5, 1 and 2 ​μg/kg) on LPS-induced alterations in level of Acetylcholine (a) and Acetylcholine-esterase activity (b) All values are Mean ​± ​SD; (n ​= ​3). ^a^p< 0.05 compared to control, ^b^p ​< ​0.05 compared to LPS group and ^c^p ​< ​0.05 compared to LPS ​+ ​DHQ-0.5 group, ^d^p ​< ​0.05 compared to LPS ​+ ​DHQ-1 group, [one-way ANOVA followed by Newman-keuls Test].

Acetylcholine is one of the dominant neurotransmitters in the brain involved in learning and memory (Myhrer, 2003). The effect of DHQ on LPS-induced alterations in the level of acetylcholine is depicted in Fig. 5(a) below. One-way ANOVA showed reckoning differences in the concentration of acetylcholine among groups [F (4, 10) ​= ​337, p ​< ​0.05]. The post-hoc test signified that LPS injection significantly brought down acetylcholine levels in the hippocampus region as compared to that of the control group. DHQ-0.5 ​μg/kg did not cause any significant change to the LPS-induced decrease in acetylcholine concentration. However, the LPS induced decrease in the acetylcholine concentration significantly increased with DHQ-1 μg/kg and DHQ-2 μg/kg. However, DHQ-2 μg/kg mediated elevation in the acetylcholine concentration was more significant than DHQ-1 μg/kg.

### 3.11 Effect of DHQ on LPS-induced changes in the acetylcholine-esterase activity

The effect of DHQ on LPS-induced changes in the AchE activity after LPS injection is depicted in Fig. 5(b) below. One-way ANOVA showed significant differences in AchE activity among groups [F (4, 10) ​= ​482, p ​< ​0.05]. It was further proved by the post-hoc test that LPS injection significantly increased the AchE activity compared to control group rats. The data further exhibited that AchE activity of the control group was also significantly lesser than all remaining groups. DHQ-0.5 ​μg/kg did not cause any significant change to the LPS-induced increase in the AchE activity. However, the LPS induced an increase in the AchE activity significantly attenuated with DHQ-1 μg/kg and DHQ-2 μg/kg. DHQ-2 ​μg/kg mediated reduction in the AchE activity was more significant than DHQ-1 μg/kg.

### 3.12 DHQ reduced pro-inflammatory IL-6 in LPS-induced animals

**Fig. 6:**
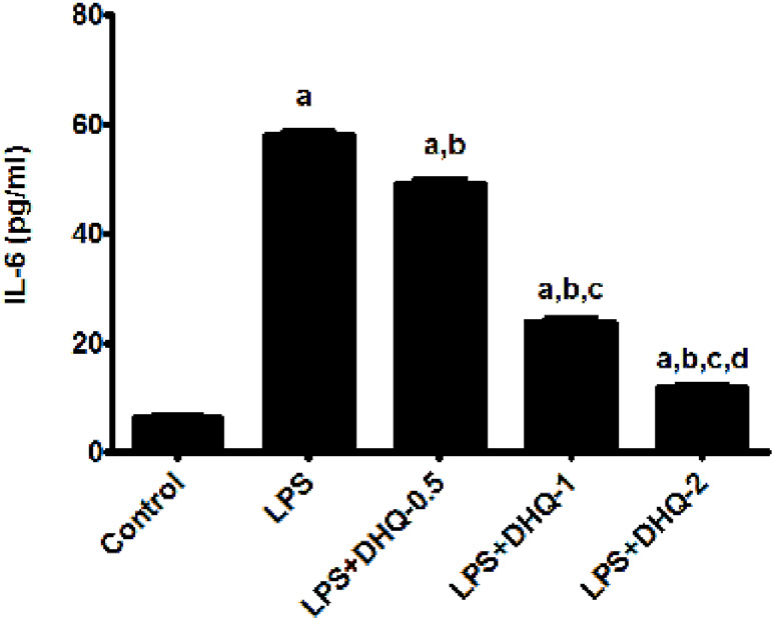
Effect of Dihydroquercetin (DHQ) -(0.5, 1 and 2 ​μg/kg) on LPS-induced alterations in level of IL-6. All values are Mean ​± ​SD; (n ​= ​3). ^a^p< 0.05 compared to control, ^b^p ​< ​0.05 compared to LPS group and ^c^p ​< ​0.05 compared to LPS ​+ ​DHQ-0.5 group, ^d^p ​< ​0.05 compared to LPS ​+ ​DHQ-1 group, [one-way ANOVA followed by Newman-keuls Test].

We studied the effect of DHQ on an inflammatory cytokine IL-6 after LPS induction as depicted in Fig. 6 below. Statistical analysis revealed significant differences in the concentration of IL-6 in the various groups. There were significant differences between the groups [F (4, 10) ​= ​3016, p ​< ​0.05] as per one-way ANOVA. The post-hoc test signified that LPS injection caused a surge in the level of IL-6 as compared to control rats. IL-6 concentration was statistically different between the groups as compared with the control group. DHQ-0.5, 1 and 2 ​μg/kg showed significant changes to the LPS-induced increase in the IL-6 level. However, DHQ-2 μg/kg mediated reduction in the level of IL-6 was the most significant when compared to the other two doses. DHQ-1 ​μg/kg was found to be more significant than DHQ-0.5 ​μg/kg.

### 3.13 DHQ showed antioxidant activities

**Fig. 7:**
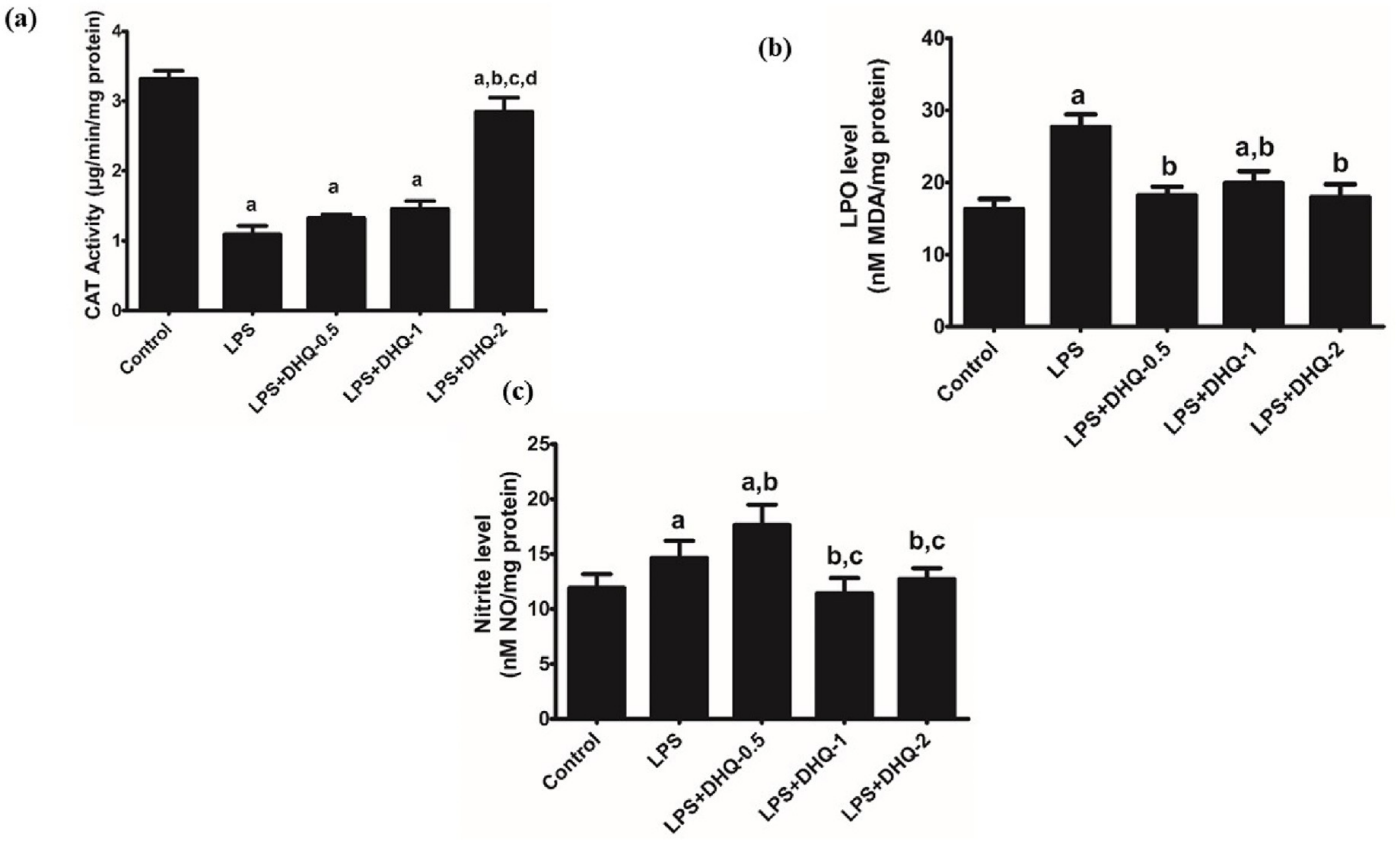
Effect of Dihydroquercetin (DHQ) -(0.5, 1 and 2 ​μg/kg) on LPS-induced alterations in anti-oxidant enzymes, (a) Catalase activity (b) LPO level (c) Nitrite level. (a) All values are mean ​± ​SD; (n ​= ​3) ^a^p<0.05 compared to control; ^b^p ​< ​0.05 compared to LPS; ^c^p ​< ​0.05 compared to LPS ​+ ​DHQ-0.5; ^d^p ​< ​0.05 compared to LPS ​+ ​DHQ-1 (one-way ANOVA followed by Student Newman–Keuls test).

We have also explored antioxidant properties of DHQ. Fig. 7 shows the effect of DHQ on the antioxidant enzymes upon LPS induction. One-way ANOVA revealed significant differences between the groups [F (4,10) ​= ​186.7, p ​< ​0.05], MDA [F (4, 10) ​= ​47.40, p ​< ​0.05] and NO [F (4, 10) ​= ​18.76, P ​< ​0.05] levels. The post-hoc test revealed that LPS injection significantly decreased CAT activity and increased LPO activity, and an increased NO level as compared with the control group. CAT activity was significantly boosted-up with DHQ-2 μg/kg administration. Moreover, LPO activity was significantly mitigated with DHQ-0.5, 1 and 2 ​μg/kg. DHQ-1 and 2 ​μg/kg but not DHQ-0.5 ​μg/kg administration mitigated NO activity significantly when compared with the LPS group.

## 4 Discussion

The salient feature of this study is that DHQ showed multimodal mechanism of actions by alleviating the LPS-induced neuroinflammation and cognitive deficits.

The relationship between neuroinflammation and learning and memory has been well established through preclinical and clinical studies (Liu et al., 2012). Y-maze is one of the commonly used mazes to evaluate learning and memory in animal models. Spontaneous alternation behavioral test is an indicative of active retrograde working memory in the Y-Maze test (Onaolapo et al., 2012). LPS decreased the alternation behavior in the Y-Maze test indicating deficits in the working memory. As shown in Fig. 2 (c) DHQ-1 and 2 ​μg/kg significantly minimized the LPS-induced loss in the working memory. It was also found that DHQ-1 and 2 ​μg/kg attenuated LPS induced decrease in the novel arm and increase in known arm entry as in Fig. 2 (b). Therefore, it improved the spatial recognition memory. The Y-maze study also showed that there was significant improvement in the spatial memory with DHQ-2 μg/kg after LPS injection as shown in Fig. 2 a. The Administration of DHQ has shown to improve spatial memory in oligomeric-Aβ-treated rodent model (Wang et al., 2018).

Previous studies (Frühauf-Perez et al., 2018) showed that LPS injection causes dysruption in the long term memory. As shown in Fig. 3 (a), DHQ in the dose of 1 and 2 ​μg/kg treatment improved the long-term memory in the TL behavioral test. As compared to day-9, TL on day-10 showed a significant reduction; this signifies an improvement in the long-term memory. An earlier study (Swiergiel and Dunn, 2007) showed that LPS injection caused anxiety-like behavior as it caused a decrease in the percentage entries as well as time spent in the open arm in the EPM study. In our study, DHQ significantly improved anxiety-like behavior as per Fig. 3 (c). LPS infusion did not show any effect on the locomotor activity of test animals as per the total arm entries data, which shows that motor functions were not hampered as in Fig. 3 (c).

Neuroinflammation decreases cerebral mean blood flow (CBF) and induces cellular stress, which could contribute to cognitive defects (Zlokovic, 2005). Its mechanism might involve dysfunction of neurovascular unit which leads to faulty clearance of neurotoxic molecules from brain to blood and an imbalance between energy metabolism and nutrition delivery (Zlokovic, 2008). In this study, LPS injection decreased the mean CBF and this decreased in mean CBF was significantly improved with DHQ-2 μg/kg, indicating its anti-neuroinflammatory activity as shown in Fig. 4.

The cholinergic system has an important role in the memory, and its imbalance is involved in the neuroinflammation pathology (Nizri et al., 2006). It has been observed that LPS induction caused an increase in AChE activity. Therefore, AChE inhibitors have been reported to ameliorate neuroinflammation-induced neurodegeneration (Nizri et al., 2006). In this study, LPS-induction caused a significant fall in the level of Ach as shown in Fig. 5 (a). and an increment in AChE activity (Fig. 5 (b)). in the hippocampus. The observed results could be due to the neuroinflammation-induced neurodegeneration of cholinergic neurons in the hippocampus (Kalb et al., 2013). DHQ-1 and 2 ​μg/kg reversed the changes caused by LPS infusion in ACh level and AChE activity as shown in Fig.5 (a) and Fig.5 (b) respectively. These data indicate that DHQ may have a beneficial effect on the cholinergic system in the neuroinflammation model of LPS.

An imbalance between ROS production and elimination in the biological system leads to oxidative stress, which plays a critical role in the age-associated cognitive decline in neuroinflammation-related neurodegenerative diseases such as Alzheimer's and Parkinson's diseases (Barnham et al., 2004). The major antioxidant enzymes, including superoxide dismutase (SOD) and catalase (CAT), are regarded as the first line of the antioxidant defense system against ROS *in vivo* during oxidative damage. SOD can convert superoxide anion to hydrogen peroxide, which is subsequently scavenged by CAT (Ighodaro and Akinloye, 2018). MDA is another well-known indicator of oxidative damage under the condition of oxidative stress (Chung et al., 2010). NO plays a crucial role in learning by facilitating long term potentiation in the hippocampus (Bon and Garthwaite, 2003), as well as involved in intracellular signaling in neurons (Feil and Kleppisch, 2008). Interestingly, an abnormal increase in reactive nitrogen species illicit apoptotic cell death by nitrosative injury to neurons (Nakagawa and Yokozawa, 2002). Further analysis revealed that LPS administration caused significant inhibition of antioxidant enzyme catalase and overactivity of certain enzymes like LPO and NO. The observed result could be due to the neuroinflammation-induced activation of ROS in the hippocampus. As shown in Fig.7 (a) and Fig.7 (b) DHQ-2 μg/kg reversed the LPS-enhanced CAT and LPO activities. LPS induced increase in NO activity was reduced by DHQ-1 and 2 ​μg/kg only Fig. 7 (c). The results showed that DHQ has an antioxidant property as reported earlier (Weidmann, 2012).

LPS is a potent activator of microglia and up-regulates the elaborations of pro-inflammatory cytokines, including IL-6 and tumor necrosis factor-α (TNF-α) (Monje et al., 2003). Our studies have shown that LPS administration caused a significant increase in the level of IL-6 in hippocampus. As shown in Fig. 6 DHQ-2 μg/kg significantly reduced the IL-6 level in the hippocampus. The results show that DHQ has an anti-neuroinflammatory activity (Sah et al., 2011). This effect of DHQ can be attributed because of its anti-oxidant activity as ROS scavenging leads to reduction in IL-6 (Chen et al., 2014; Jain et al., 2009).

In the future perspectives, effect of DHQ on LPS and amyloid beta induced activation of NF-kappa B via toll like receptor-4 activation in the microglial cells can be explored as NF-kappa B is one of the potential targets for neuroinflammation and related complications like dementia (Kim et al., 2015).

## 5 Conclusion

DHQ has improved LPS-induced memory impairment and anxiety like behavior in the rat. It has minimized LPS-induced cholinergic dysfunction in the hippocampus. It has also shown to possess antioxidant activity. DHQ has shown anti neuroinflammatory activity by reducing IL-6 a prominent cytokine involved in neuroinflammation. Hence, preclinical evidence points to DHQ as a potential candidate in the management of neuroinflammation and other neurodegenerative disorders involving memory impairment.

## Author contribution

SK and QA conceived and designed the study. QA was responsible for acquisition of data. SK and QA analyzed and interpreted the data. SK and QA drafted the work for intellectual content and context. SK did the final approval and takes overall responsibility of the published work.

## Declaration of competing interest

The authors declare that they have no known competing financial interests or personal relationships that could have appeared to influence the work reported in this paper.
